# Tocilizumab in severe COVID-19 – A randomized, double-blind, placebo-controlled trial

**DOI:** 10.1016/j.imj.2022.05.001

**Published:** 2022-06-02

**Authors:** Muhammad Irfan Malik, Sardar Al Fareed Zafar, Fabiha Qayyum, Muna Malik, Muhammad Sohaib Asghar, Muhammad Junaid Tahir, Ammarah Arshad, Fatima Khalil, Hafiza Shafia Naz, Mudassar Aslam, Jodat Saleem, Abdul Aziz, Mustafa Usman Azhar, Muhammad Naqash, Zohaib Yousaf

**Affiliations:** a Postgraduate Medical Institute, Lahore, Pakistan; b Lahore General Hospital, Lahore, Pakistan; c Ameer-ud-Din Medical College, Lahore, Pakistan; d Dow University of Health Sciences–Ojha Campus, Karachi, Pakistan; e Sheikh Zayed Medical College, Rahim Yar Khan, Pakistan; f Hamad Medical Corporation, Doha, Qatar

**Keywords:** Inflammatory, Infectious, Immunosuppression, COVID-19, Therapy, Trial

## Abstract

**Background:**

The therapeutic effectiveness of interleukin-6 receptor inhibitor in critically ill hospitalized patients with coronavirus disease 2019 (COVID-19) is uncertain.

**Methods:**

To evaluate the efficacy and safety of the outcome as recovery or death of tocilizumab for severe acute respiratory syndrome-coronavirus-2 (SARS-CoV-2) infection, we conducted a randomized, double-blinded, placebo-controlled phase 2 trial in critically ill COVID-19 adult patients. The patients were randomly assigned in a 4:1 ratio to receive standard medical treatment plus the recommended dose of either tocilizumab or the placebo drug. Randomization was stratified. The primary outcome was the recovery or death after administration of tocilizumab or a placebo drug. The secondary outcomes were clinical recovery or worsening of the patients' symptoms and inflammatory markers and discharge from the hospital.

**Results:**

Of 190 patients included in this study, 152 received tocilizumab, and 38 received a placebo. The duration of hospital stay of the interventional group was 12.9 ± 9.2, while the placebo group had a more extended hospital stay (15.6 ± 8.8). The mortality ratio for the primary outcome, ie, mortality or recovery in the tocilizumab group was 17.8%; *p* = 0.58 by log-rank test). The mortality ratio in the placebo group was 76.3%; *p* = 0.32 by log-rank test). The inflammatory markers in the tocilizumab group significantly declined by day 16 compared to the placebo group.

**Conclusions:**

The use of tocilizumab was associated with decreased mortality, earlier improvement of inflammatory markers, and reduced hospital stay in patients with severe COVID-19.

## 1 Introduction

Coronaviruses are RNA viruses. Six coronaviruses are pathogenic to humans, mainly causing respiratory tract infections. Severe acute respiratory syndrome-coronavirus (SARS-CoV) in 2003 and the middle east respiratory syndrome-coronavirus (MERS-CoV) in 2012 were responsible for outbreaks of severe respiratory tract infections with 11%–35% mortality [1]. In December 2019, a novel beta coronavirus SARS-CoV-2 causing coronavirus disease 2019 (COVID-19) was discovered, and on March 11, 2020, the World Health Organization (WHO) declared it as a pandemic [2,3].

Mortality in COVID-19 is associated with older age, male gender, comorbidities like coronary artery disease, diabetes mellitus, hypertension, chronic lung or renal diseases, and an immunocompromised state [4,5]. The case fatality rate (CFR) of COVID-19 is 2.3%, and the percentage increases to 10% with cardiovascular and pulmonary illness and 26% to 88% in intubated patients [6]. The leading cause of death in patients with severe COVID-19 is acute respiratory distress syndrome (ARDS) combined with disseminated intravascular coagulation [6].

Severe COVID-19 is responsible for the production of proinflammatory cytokines. The autopsy showed that severe disease is associated with deviant host-immune response and cytokine storm, resulting in cytokines release syndrome (CRS) [7]. The over-activation of the immune system causes multiple derangements in laboratory parameters like cytopenia, increased D-dimers, raised serum ferritin, and transaminitis [8]. Elevated inflammatory biomarkers like C-reactive protein (CRP) and proinflammatory cytokines like interleukin-2 (IL-2), interleukin-6 (IL-6), interleukin-10 (IL-10), gamma interferon (INF), and tumor necrosis factor-alpha (α-TNF) are higher in severe COVID-19 [9,10]. Early use of IL-6 inhibitors is associated with a decrease in morbidity and mortality [12]. As cytokine storm has no unified clinical definition, we considered any patient with raised inflammatory markers and evidence of cellular damage as suffering from cytokine storm.

Tocilizumab is a monoclonal antibody, an IL-6 receptor inhibitor commonly used to treat rheumatoid arthritis [13]. Food and Drug Administration approves this drug for Emergency Use Authorization for the treatment of COVID-19 in hospitalized patients >2 years of age who are receiving systemic corticosteroids and require supplemental oxygen, noninvasive or invasive mechanical ventilation (IMV), or extracorporeal membrane oxygenation. Literature shows clinical benefits in severe COVID-19 [14,15]. This study aims to assess the role of tocilizumab in hospitalized patients with severe COVID-19. We hypothesize that the use of tocilizumab and standard treatment for severe COVID-19 will result in better overall survival and a shorter hospital stay compared to no tocilizumab in an inpatient population.

## 2 Materials and Methods

### 2.1 Study design

This is a single-center, randomized, double-blind, placebo-controlled phase 2 trial. All patients received treatment according to the local protocol at the hospital. In addition, tocilizumab or placebo was administered to the patients enrolled in the study.

### 2.2 Study settings

The study was conducted in Lahore General Hospital, Lahore, Pakistan, from May 3, 2020 to October 3, 2020.

### 2.3 Ethical consideration

Ethical approval was obtained from the research ethical committee of Lahore General Hospital, having approval number 00–144–20 (ClinicalTrials.gov ID: NCT04560205). The study was conducted in full conformance with the Declaration of Helsinki, Drug Regulatory Authority of Pakistan, Good Clinical Practice, and as per the regulations of the Pakistan Health Research Council. Informed consent was obtained from all patients prior to inclusion.

### 2.4 Sample size and methodology

Lahore General Hospital is a 1600 bedded tertiary care hospital. Among 1600 beds, 150 beds were allocated to COVID-19 patients. These beds were of varying acuity from the medical floor to intensive care units and high dependency units. The sampling technique was consecutive sampling. A total of 1220 in-hospital suspected patients underwent nasopharyngeal SARS-CoV-2 real-time-polymerase chain reaction (RT-PCR) screening employing Zeesan viral RNA (ribonucleic acid) extraction kit. A total of 679 swabs were positive. Of 323 patients fulfilling the inclusion criteria, 190 patients consented to be included in the study. Of those 190 patients, 152 received tocilizumab, and 38 received a placebo. We estimated this sample size by presuming that the patients' risk of IMV or their death during hospitalization would be 56% in the placebo group, which would drop by 40% in patients treated with tocilizumab as reported by Petrak et al. [16].

### 2.5 Randomization

Simple randomization was done. The patients were randomly assigned in a 4:1 ratio to receive standard medical treatment plus the recommended dose of either tocilizumab or the placebo. After the consent and randomization, administration of tocilizumab or placebo was completed within 2 hours.

#### 2.5.1 Inclusion criteria

• Age 18–75 years
• Patients of any gender.
• COVID-19 was diagnosed by nasopharyngeal RT-PCR for SARS-CoV-2
• Hospitalization secondary to COVID-19 at the time of enrolment.
• Pneumonia with typical radiological changes of COVID-19 on X-ray chest or high-resolution computed tomography chest.
• Cytokine storm as evidenced by raised inflammatory markers.

Inclusion criteria was the same for the patients >55 years and patients <55 years with comorbidities. Patients with <55 years age and no comorbidities had a different inclusion criterion as mentioned below:

1 *Age <55 years (with no comorbidities):*
  ○ The patient is unable to maintain O_2_ saturation > 93% with 7–10 liter of oxygen, Persisting tachypnea beyond >35/ min
  ○ ≥2 biochemical markers (lymphocyte count, CRP, LDH, D-dimer, serum ferritin) are increasing by > 50% of the upper limit of normal

i.e., CRP >50 mg/L
LDH >1000 U/L
D-dimer >1 mg/L or 1000 ng/mL
Serum ferritin >1000 ng/mL or mcg/L
Interleukin-6 (IL-6) <7 pg/mL

1 *Age >55 years (with or without comorbidities) or <55 years (with comorbidities):*
  ○ The patient is unable to maintain O_2_ saturation >93% with 5 liters of oxygen.
  ○ Tachypnea >30/min.
  ○ ≥2 elevated biochemical markers (lymphocyte count, CRP, lactate dehydrogenase (LDH), D-dimer, serum ferritin) are increasing by > 50% from the upper limit of normal, i.e., CRP > 50 mg/L, LDH >1000 U/L, D-dimer > 1 mg/L or 1000 ng/mL, Serum ferritin >1000 ng/mL or mcg/L, Interleukin-6 (IL-6) <7 pg/mL.

#### 2.5.2 Exclusion criteria

• Patients who do not require supplemental oxygen.
• Patients on IMV at the time of enrolment.
• Patients with respiratory rate <30/mins.
• Patients with lymphocyte count, CRP, LDH, D-dimer, serum ferritin, or IL-6 not deranged >50% (any two of these parameters deranged >50%).
• Patients with active tuberculosis (TB).
• Patients suffering from Herpes zoster.
• Patients who have multiple sclerosis.
• Allergy to tocilizumab.
• Presences of chronic renal failure >4 stage, GFR <30 mL/min/1.73m^2^.
• Alanine aminotransferase/aspartate aminotransferase >5 times than normal values.
• Presences of neutropenia <500/mm^3^.
• Platelets count less than 50 ×10^3^ /µL.
• Complicated diverticulitis/ intestinal perforation.
• Immune-suppressive antirejection therapy.
• Pregnancy/breastfeeding.
• Previous myocardial infarction/ischemic heart disease, heart failure.
• Psychiatric patients.

### 2.6 Variables

The information collected included patients’ demographics, presenting symptoms, disease severity, comorbidities, including the history of smoking, hypertension, diabetes mellitus, asthma, chronic obstructive pulmonary disease, tuberculosis, chronic hepatitis B or C, and chronic kidney disease. Laboratory tests in which complete blood count, liver function tests, renal function tests, CRP, LDH, serum ferritin, D-dimer, and IL-6 were recorded on the day of admission, followed by the third day, seventh day and sixteenth day of admission. The parameters above were monitored before initiating tocilizumab. Vital signs were recorded both pre- and postintervention with tocilizumab or placebo. The dose of the drug, its duration, and outcomes, including the length of hospital stay, complications, and mortality, were abstracted on the approved case record form.

### 2.7 Standard care

Standard medical treatment was provided to all the patients. The standard of care included a combination of intravenous antibiotic (ceftriaxone), methylprednisolone (1–2 mg/kg as a loading dose followed by 0.5–1 mg/kg twice daily), and enoxaparin sodium (40 mg subcutaneous once daily). If the patient did not respond, the dose of methylprednisolone was increased to 1.5 times as loading dose followed by twice daily.

### 2.8 Placebo group

As an adjuvant to the standard medical treatment, a placebo was administered intravenously. The placebo consisted of a normal saline infusion administered over 60 minutes. The infusion was administered at the patient's bedside in a look-alike bag similar to the tocilizumab infusion.

### 2.9 Treatment group

As an adjuvant to the standard medical treatment, 4–8 mg/kg with 800 mg maximum dose of tocilizumab was given in 60 minutes intravenous infusion, followed by up to 2 additional doses according to response (clinical as well as laboratory parameters) after the first dose. The tocilizumab infusion was prepared prior to bringing it to the bedside to keep the blinding intact.

### 2.10 Outcomes

The primary outcomes were recorded as either recovery or death of the patient after administering tocilizumab or placebo. The primary outcome was assessed as survival function and hazard function. The secondary outcomes were recorded as clinical recovery or worsening of symptoms of the patients and their inflammatory markers (at day 0, 2, 4, 8, and 16), their progression from non-IMV to IMV, escalation of care to the ICU or discharge from hospital. The secondary outcomes included clinical biomarkers (lymphocyte count, CRP, LDH, D-dimer, serum ferritin, and IL-6) and their association with the demographics and efficacy outcomes.

### 2.11 Statistical analysis

#### 2.11.1 Data collection

Data was entered into a case record form and was subsequently accessed by study investigators before being exported into a study-specific excel spreadsheet. Each entry was coded using a unique patient identifier, accessible only to investigators. Links between the names and the unique patient identifier codes were kept in an Excel sheet locked within the principal investigator's (PI) computer and were destroyed post completion of analysis.

#### 2.11.2 Data analysis

Data were analyzed by SPSS version 25.0. Having 190 patients, we had 80% power to find the difference between two groups using the log-rank test, assuming the two-sided tests with a significance level of 0.05. The primary outcome was either recovery or death, ensuring that all the patients included in the study were administered with either tocilizumab or the placebo drug and assessing their survival plot. The difference between the groups being treated was estimated by hazard plotting. The survival and the hazard were analyzed using Kaplan-Meier curves with a 95% confidence interval.

## 3 Results

### 3.1 Demographics

**Fig. 1:**
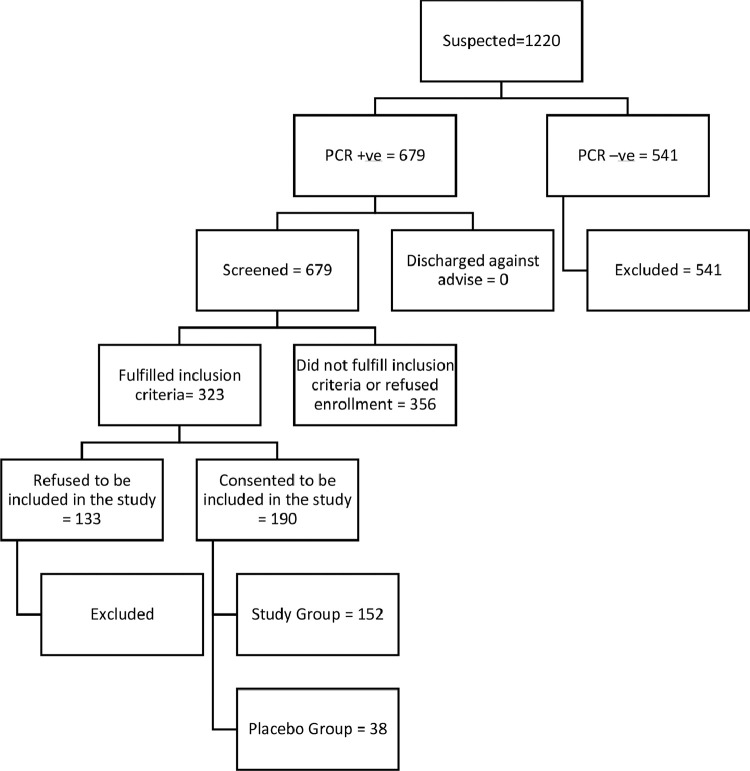
Screening, randomization, and enrollment of the patients. PCR, polymerase chain reaction.

Between May 3, 2020 and October 3, 2020, 190 patients were enrolled. A total of 152 patients received tocilizumab, and 38 received a placebo (Fig. 1). The patients' demographics are shown in Table 1. A total of 102 (67.1%) were male in the control group, and 50 (32.9%) were female. 29 (76.3%) were male in the placebo group, and 9 (23.6%) were female. The mean age in the tocilizumab group was 45.6 ± 12.2 years, and for the placebo group, it was 44.6 ± 13.7 years.

**Table 1:** Demographic and disease characteristics of the patients at baseline.

		Tocilizumab			Placebo	
	Characteristic	(n = 152)			(n = 38)	
	Gender					
	Male sex — no. (%)	102	(67.1)		29	(76.3)
	Female sex –- no. (%)	50	(32.9)		9	(23.6)
	Age					
	Mean — years	45.6 ± 12.2			44.6 ± 13.7	
	Distribution — no. (%)					
	18–64 years	137	(90.1)		28	(77.7)
	65–84 years	15	(9.8)		8	(22.2)
	Sequential organ failure assessment score SOFA score 2+	39(25.7)			3(7.8)	
	Noninvasive mechanical ventilation					
	Patients — no. (%)	101	(66.4)		6	(15.7)
	Lung involvement in chest X-ray					
	<50% Lung involvement/infiltrates	18	(11.8)		19	(50.0)
	>50% Lung involvement/infiltrates	134	(88.1)		19	(50.0)
	Patient mortality	27		(17.7)	29	(76.3)
	Tocilizumab doses – no. (%)					
	1 Dose	33		(21.7)	–	–
	2 Doses	88		(57.9)	–	–
	3 Doses	31		(20.4)	–	–
	Day of first intervention of tocilizumab (from the day of admission)					
	Mean — Days	2.4 ± 2.9			–	–
	Duration of hospital stay					
	Mean — Days	12.9 ± 9.2			15.6 ± 8.8	
	Comorbidities					
	Smoking — no. (%)	11		(7.2)	2	(5.3)
	Hypertension — no. (%)	66		(43.4)	8	(21.1)
	Diabetes mellitus — no. (%)	81		(53.3)	11	(28.9)
	Asthma — no. (%)	10		(6.6)	0	(0.0)
	aCOPD — no. (%)	8		(5.3)	1	(2.6)
	Tuberculosis — no. (%)	2		(1.3)	0	(0.0)
	Hepatitis B/C — no. (%)	6		(3.9)	0	(0.0)
	Others — no. (%)	21		(13.8)	1	(2.6)

a COPD = chronic obstructive pulmonary disease

Maximum three doses of tocilizumab were given to the patients; 88 patients received 2 doses. A total of 88% of the patients (n = 133) presented with >50% lung infiltration in interventional group. However, half of the patients presented with <50% lung involvement in the placebo group. The duration of hospital stay of the interventional group was 12.9 ± 9.2 days. While the placebo group had comparatively longer hospital stays of 15.6 ± 8.8 days. Diabetes mellitus was the most common comorbid condition in interventional 81 (53.3%) and placebo group 11 (28.9%).

A total of 27 (17.7%) patients treated with tocilizumab were intubated and later died. In the placebo group, of 29 (6.3%) patients who died, 21 (N = 38, 55.2%) were intubated, and 8 (21%) patients died without intubation. The rest of the results are shown in Table 1.

### 3.2 Survival analysis

**Fig. 2:**
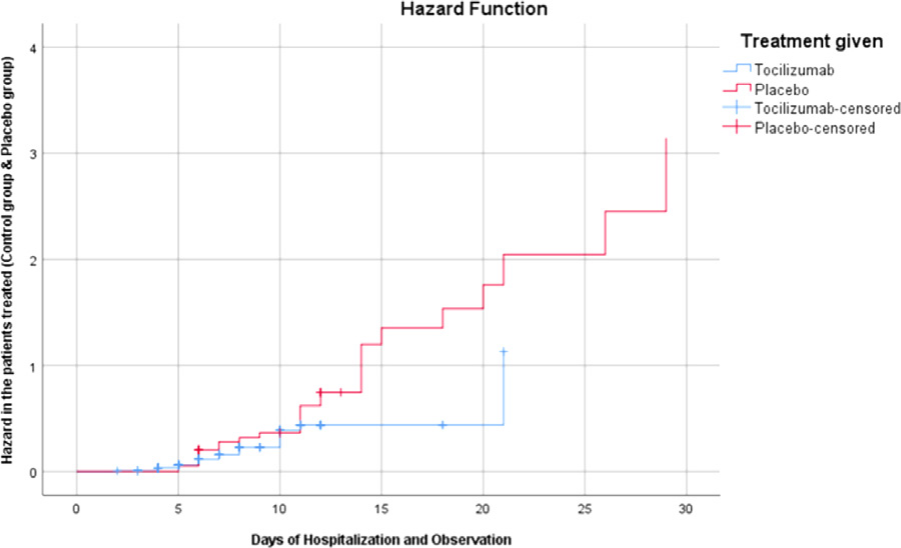
Overall Hazard curves from baseline to day 30 in tocilizumab patients according to Treatment given.

**Fig. 3:**
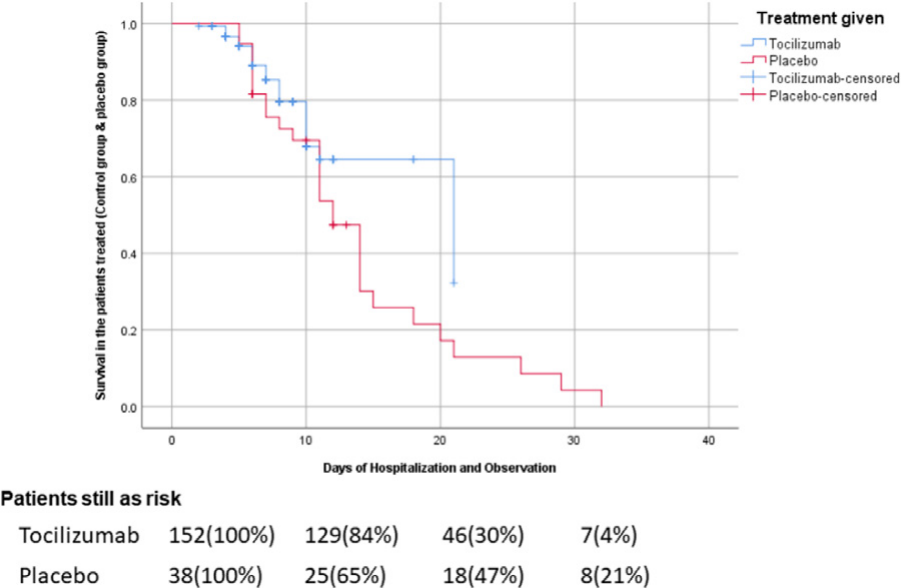
Overall Survival curves from baseline to day to in tocilizumab patients according to treatment given.

The Kaplan-Meier curves for the survival analysis in the patients receiving tocilizumab and the placebo are shown in Fig. 2 and Fig. 3. The days of hospitalization and patient under observation were taken at the x-axis as an independent variable. The relative survival and hazard in both groups (tocilizumab and placebo) were taken at the y-axis as a dependent variable. The mortality ratio for the primary outcome, ie, mortality or recovery in the tocilizumab group was 17.8%; *p*= 0.58 by log-rank test). The mortality ratio in the placebo group was 76.3%; *p*= 0.32 by log-rank test).

### 3.3 Laboratory investigation trend

The laboratory investigations of the 152 patients receiving tocilizumab and 38 patients receiving placebo drug were followed up (at day 0, 2, 4, 8, 16). The trend of the laboratory investigations in the follow-up time of 16 days since the start of interventions was analyzed (Table 2). The correlation of the laboratory investigations with the number of doses of tocilizumab needed to be administered is also mentioned in Table 2. The most significant factor to be followed with tocilizumab dosage was IL-6 levels. IL-6 was found to be in inverse relation with the number of doses of tocilizumab administered. The biomarkers in the tocilizumab group were significantly reduced at day 16. The trend of laboratory investigations in the placebo group showed lesser variability than tocilizumab, as mentioned in Table 2.

**Table 2:** Laboratory investigations follow up of patients.

	Tocilizumab		Pearson's correlation with No. of Tocilizumab doses administered	Placebo	
Characteristic	(n = 152)		*r*	(n = 38)	
Lymphocytes – 10^3 cells/µL					
Before intervention	10.5	± 8.1	−0.003	12.6	± 9.2
Day 2 after intervention	9.7	±6.4	0.53	9.0	± 4.9
Day 4 after intervention	8.5	± 4.9	0.277	9.0	± 3.7
Day 8 after intervention	7.7	± 4.0	0.152	11.3	± 4.5
Day 16 after intervention	3.6	± 2.5	0.190	14.4	± 5.2
Lactate dehydrogenase — units/L					
Before intervention	957.3	± 556.5	−0.146	1191.6	± 623.7
Day 2 after intervention	1081.9	± 560.0	−0.058	1135.9	± 530.6
Day 4 after intervention	1123.1	± 631.2	−0.260	1057.9	± 630.1
Day 8 after intervention	864.7	± 417.7	−0.046	968.8	± 624.2
Day 16 after intervention	527.9	± 304.2	−0.028	1129	± 531.1
Interleukin-6 — pg/mL					
Before intervention	505.4	± 967.6	−0.129	428.2	±379.5
Day 2 after intervention	477.7	± 826.6	0.377	622.8	±442.1
Day 4 after intervention	492.2	± 662.2	−0.009	857.9	±381.7
Day 8 after intervention	264.4	± 456.4	−0.267	993.9	±637.2
Day 16 after intervention	62.5	± 45.0	−0.369	847	±396.8
C-reactive protein — mg/liter					
Before intervention	29.9	± 40.0	−0.461	17.0	± 11.7
Day 2 after intervention	26.6	± 30.6	−0.459	20.1	± 13.9
Day 4 after intervention	22.8	± 43.9	−0.389	18.2	± 17.9
Day 8 after intervention	11.4	± 18.4	−0.414	11.2	± 13.2
Day 16 after intervention	5.6	± 7.6	−0.414	11.7	± 7.5
D-Dimer — mg/mL					
Before intervention	2.8	± 3.8	−0.093	3.6	± 3.4
Day 2 after intervention	2.3	± 2.2	0.243	3.7	± 3.1
Day 4 after intervention	2.4	± 2.4	−0.162	3.0	± 3.4
Day 8 after intervention	1.8	± 2.6	−0.154	3.3	± 4.0
Day 16 after intervention	1.0	±1.5	0.035	4.4	± 3.8
Ferritin — ng/mL					
Before intervention	857.6	± 585.6	−0.062	903.2	± 536.5
Day 2 after intervention	758.2	± 504.9	0.041	914.4	± 557.7
Day 4 after intervention	718.6	± 598.3	0.010	736.3	± 622.3
Day 8 after intervention	618.6	± 452.6	−0.009	596.5	± 499.9
Day 16 after intervention	438.0	± 322.0	0.001	895.2	± 549.3

## 4 Discussion

Tocilizumab is used for cytokine storm associated with COVID-19 [15,17]. In this study, we observed 190 COVID-19 patients with laboratory-confirmed SARS-CoV-2 infections. 158 patients were treated with tocilizumab, and 32 were treated with a placebo.

The majority of the patients of this study had more than 50% lung infiltrates, which is also consistent with the study conducted by Zhang et al. [18]. Mechanical ventilation is required to manage ARDS cases of COVID-19 disease; 66% of the patients treated with tocilizumab required non-invasive ventilation, and 15% of placebo group patients required non-invasive ventilation.

In a study of severe COVID-19 patients, the clinical data presented that most patients presented with respiratory symptoms, hypoxemia, and radiographic changes recovered clinically after tocilizumab treatment [19]. The use of tocilizumab has also been documented in other studies claiming faster improvement with oxygenation and decreased CRP levels in critically ill COVID-19 patients [20,21].

In this study, the laboratory markers of COVID-19 patients were followed on the day of admission and then on days 2, 4, 8, and day 16 of the intervention. We observed that the inflammatory markers improved markedly on the 16th day of tocilizumab administration. These findings are consistent with the study conducted by Zheng et al. [19].

The usage of 2 or 3 doses of tocilizumab is still debatable. In this study, most patients received two doses of tocilizumab. Patients with partial response to 2 doses were administered a third dose of tocilizumab [22]. This impacted the prognostic significance of tocilizumab as a better response was shown by the patients receiving three doses of tocilizumab who previously remained unresponsive to 2 doses. The survival analysis and Hazard analysis in this study suggested the benefit of tocilizumab as hospital stays among the patients receiving tocilizumab turned out to be 12.9 ± 9.2 days. However, the Log-Rank test in the Kaplan-Meier curve failed to reach a statistical significance. This is likely due to the small sample size being unable to power the study adequately.

In the patients receiving placebo drugs, it turned out to be 15.6 ± 8.8 days. The reduction in the days of hospitalization has been observed in a previous study published in The New England Journal of Medicine (NEJM). This study confirmed the better prognostic outcome of tocilizumab as the critical care and days under observation were reduced to quite optimum levels [23]. As tocilizumab reduces the immune response, it could also lead towards expectations of a more significant number of infections in this group [21]. So more focused monitoring of infections is needed in the patients' receiving tocilizumab. This study engenders a stimulating hypothesis of the safety and efficacy of tocilizumab drugs in critically-ill COVID-19 patients.

The strength of this study lies in its design being prospective, randomized, double-blind, and placebo-controlled. However, the simple randomization eliminates further matching in the two groups beyond the inclusion criteria. This randomization method has led to a mismatch in certain baseline characteristics in the two groups. The tocilizumab group has a higher percentage of subjects with >50% lung involvement on chest X-ray (88.1%) as compared to the placebo group (50%). Also, even though the mean age and standard deviations are comparable between the two groups, the subgroup of age 65–84 is greater in the placebo group than the tocilizumab group (22.2% vs 9.8%).

## Author contributions

M.I.M, S.A.F.Z, M.M, J.S, and F.Q conceived the idea; F.Q, M.N, M.I.M, A.A (Ammarah Arshad), A.A (Abdul Aziz), F.K, M.A, S.A.F.Z, J.S, and M.M collected the data; F.Q, M.S.A, and Z.Y analyzed and interpreted the data; F.Q, M.M, M.J.T, M.S.A, Z.Y, H.S.N, A.A (Abdul Aziz), A.A (Ammarah Arshad), M.U.A, and F.K did write up of the manuscript; and finally, Z.Y, M.S.A, M.J.T, and M.I.M reviewed the manuscript for intellectual content critically. All authors approved the final version of the manuscript.

## Declaration of competing interests

The authors declare that they have no known competing financial interests or personal relationships that could have appeared to influence the work reported in this paper.

## Funding sources

This research did not receive any specific grant from funding agencies in the public, commercial, or not-for-profit sectors.

## Data avabliable statment

The patient information that supports the findings of this study are available on request from the corresponding author. The data are not publicly available due to privacy or ethical restrictions.

## Ethical statement

Ethical approval was obtained from the research ethical committee of Lahore General Hospital, having approval number 00-144-20 (ClinicalTrials.gov ID: NCT04560205). The study was conducted in full conformance with the Declaration of Helsinki, Drug Regulatory Authority of Pakistan, Good Clinical Practice, and as per the regulations of the Pakistan Health Research Council. Informed consent was obtained from all patients prior to inclusion.
